# No fitness cost associated with Asn-2041-Ile mutation in winter wild oat (*Avena ludoviciana*) seed germination under various environmental conditions

**DOI:** 10.1038/s41598-021-81310-8

**Published:** 2021-01-15

**Authors:** Saeid Hassanpour-bourkheili, Javid Gherekhloo, Behnam Kamkar, S. Sanaz Ramezanpour

**Affiliations:** 1 Agronomy Department, Plant Production Faculty, Gorgan University of Agricultural Sciences and Natural Resources, Gorgan, Iran; 2 Agronomy Department, Agriculture Faculty, Ferdowsi University of Mashhad, Mashhad, Iran; 3 Plant Breeding and Biotechnology Department, Plant Production Faculty, Gorgan University of Agricultural Sciences and Natural Resources, Gorgan, Iran

**Keywords:** Plant sciences, Plant ecology

## Abstract

Knowledge about the fitness cost imposed by herbicide resistance in weeds is instrumental in devising integrated management methods. The present study investigated the germination response of ACCase-resistant (R) and susceptible (S) winter wild oat under different environmental conditions. The DNA of the plants was sequenced after being extracted and purified. The segregated F_2_ seeds were subjected to various temperatures, water potentials, NaCl concentrations, different pHs, darkness conditions, and burial depths. The results of the sequencing indicated that Ile-2041-Asn mutation is responsible for the evolution of resistance in the studied winter wild oat plants. The seeds were able to germinate over a wide range of temperatures, osmotic potentials, NaCl concentrations, and pHs. Germination percentage of R and S seeds under dark and light conditions was similar and ranged from 86.3 to 88.3%. The highest emergence percentage for both R and S plants was obtained in 0, 1, and 2 cm depths and ranged from 66.6 to 70.3%. In overall, no differences were observed in the germination response between the R and S winter wild oat plants under all studied conditions. No fitness cost at seed level indicates that control of R winter wild oats is more difficult, and it is essential to adopt crop and herbicide rotation to delay the further evolution of resistance.

## Introduction

Consecutive application of herbicide has led to the evolution of herbicide-resistant weeds, a major threat to the sustainable production of agricultural crops^1^. Herbicide-resistant plants may outcompete the susceptible ones under herbicide selection pressure, however, this resistance may impose a fitness cost on the plant^2^. Fitness cost may be defined as the decline in relative fitness of the plant due to direct or pleiotropic effects imposed by the resistance alleles^3^. Herbicide resistance does not necessarily impose fitness cost^4,5^, i.e., this cost is not inevitably negative and the mutation may even enhance the resistant plants^6^. Also, each mechanism of resistance (e.g. different gene mutations) may impose a different fitness cost on the plant^2^.

Investigation of fitness cost associated with herbicide resistance may be performed via various plant traits including vegetative characteristics, yield, fecundity, phenology, and seed germination, with the latter described as one of the most important components of plant fitness^7^. Seed germination is a highly important stage in the life cycle of plants which may determine the success of the plant from the establishment to maturity^8^. This process is influenced by various environmental factors such as temperature^9^, drought^10^, salinity^11^, pH^12^, light^13^, burial depth^14^, etc.

The difference between the germination response of herbicide-susceptible and -resistant weeds has been studied in numerous weeds such as Japanese foxtail (*Alopecurus japonicus* L.)^15^, littleseed canarygrass (*Phalaris minor* Retz.)^16^, sourgrass (*Digitaria insularis* (L.) Fedde)^17,18^, American slough grass (*Beckmannia syzigachne* Steud. Fernald)^19^, kochia (*Bassia scoparia* (L.) A.J. Scott)^20^ and wild oats (*Avena* spp.)^21,22^.

Winter wild oat (*Avena ludoviciana* Dur.) is one of the most troublesome weeds widely distributed in various parts of the world including the Caucasus^23^, the Himalayas^24^, South and North America, Africa, the Middle East, and most of Europe^25^. This weed infests various cereals and legumes such as oilseed rape, and the application of herbicides is a very common option for its control^26^. However, consistent selective pressure imposed by herbicide application has resulted in numerous cases of resistance in this weed, with most cases being associated with acetolactate synthase (ALS) and acetyl coenzyme A carboxylase (ACCase) inhibitors^27^.

Some researchers have assessed the costs of different resistance mechanisms on winter wild oat. There are also papers on the relative fitness of wild oat (*Avena fatua* L.)^28,29^. In a study regarding the fitness cost of resistance to ACCase inhibitors in winter wild oat, it was revealed that both resistant and susceptible populations had almost similar germination growth under competitive and non-competitive^22^. ACCase-resistant winter wild oat populations collected in Greece with Trp-1999-Cys, Trp-2027-Cys, Ile-2041-Asn, Asp-2078-Gly, and Cys-2088-Arg mutations in their *ACCase* encoding genes presented similar growth under field conditions than susceptible population, but the researchers also reported inconsistency in the fitness difference between the susceptible and resistant populations which may be due to other non-resistance traits^30^. Winter wild oat populations with Asn-2041-Ile mutation in *ACCase* encoding gene has been described by Hassanpour-bourkheili^31^, with resistance to haloxyfop-R methyl ester, clodinafop propargyl, and sethoxydim.

Knowledge about the fitness cost of herbicides is instrumental in the development of integrated weed management methods to overcome herbicide resistance^2^. However, only a few studies are available on the germination response of ACCase-resistant weeds under environmental conditions. Moreover, no reports are available on the impact of resistance to ACCase inhibitors on the germination of winter wild oat under environmental conditions. Thus, the present study will quantify the germination response of ACCase-resistant (R) winter wild oat compared to the susceptible (S) biotype under various environmental conditions.

## Results

### Sequencing of the *ACCase* gene

**Figure 1:**
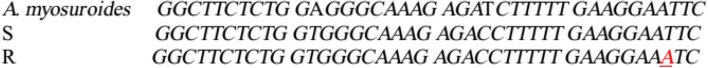
Sequencing results for winter wild oat biotypes along with *A. myosuroides*. 2041 point is highlighted in red.

*ACCase* gene sequencing results confirmed that only Ile-2041-Asn mutation conferred resistance to ACCase inhibitors in the R winter wild oat biotype (Fig. 1), and no other mutation (Table 1) was observed.

**Table 1:** Primers used for sequencing of the acetyl coenzyme A carboxylase gene in resistant (R) and susceptible (S) winter wild oat biotypes.

Primer	Sequence	Annealing temperature (°C)	Assay
SQCT-α1F	AATACATGTGATCCTCGTGCAG	63	1999–2096 sequencing
SQCT-α 1R	TCCTCTGACCTGAACTTGATCTC	63	1999–2096 sequencing
SQCT-β1F	CATCATCTTTCTGTATGCCAGTGGG	65	1781 sequencing
SQCT-β 1R	CTGTATGCACCGTATGCCAAG	65	1781 sequencing

The amino acid numbering follows the *Alopecurus myosuroides* chloroplastic *ACCase* sequence (Genbank biotype no. AJ310767).

### Temperature assay

The highest germination percentages for both R and S winter wild oat biotypes were 87.3 and 89.0%, respectively, which were observed at 25 °C. The lowest D_50_ (time to 50% germination) value estimated_,_ also recorded at 25 °C, was at 34.7 and 36.2 h for S and R biotypes, respectively, and was there were no germinate at 37 °C. There was no difference between S and R biotypes regarding the estimated parameters (Table 2). Because the highest germination for both biotypes was observed at 25 °C, the germination response of the biotypes under the studied environmental conditions was conducted at this temperature.

**Table 2:** Parameter estimates resulted from the fitting of three-parameter sigmoidal function to seed germination data associated with herbicide-resistant (R) and -susceptible (S) winter wild oat biotypes over time under different temperatures. The values in parentheses denote standard error.

Temperature (°C)	Parameter							
	*Gmax* (%)		*b*		*X*_50_ (h)		*D*_50_ (h)	
	S	R	S	R	S	R	S	R
5	51.8 (2.1)^a^	50.9 (1.8)^a^	23.5 (3.1)^a^	21.0 (2.6)^a^	82.9 (4.5)^a^	80.4 (3.9)^a^	161.5 (8.5)^a^	164.5 (10.2)^a^
10	60.2 (1.3)^a^	59.3 (1.3)^a^	13.1 (1.5)^a^	13.5 (1.6)^a^	49.9 (1.9)^a^	52.5 (2.0)^a^	70.8 (6.9)^a^	74.9 (7.2)^a^
15	77.2 (1.8)^a^	75.4 (1.4)^a^	9.6 (1.5)^a^	9.3 (1.2)^a^	41.2 (1.6)^a^	42.2 (1.3)^a^	47.1 (4.3)^a^	48.5 (4.6)^a^
20	84.5 (2.0)^a^	82.7 (1.9)^a^	9.5 (1.4)^a^	10.3 (1.4)^a^	35.7 (1.5)^a^	36.9 (1.5)^a^	39.2 (3.8)^a^	41.3 (4.1)^a^
25	89.0 (1.4)^a^	87.3 (1.2)^a^	7.6 (1.0)^a^	7.8 (1.1)^a^	32.8 (1.3)^a^	33.9 (1.3)^a^	34.7 (3.8)^a^	36.2 (3.9)^a^
30	75.6 (1.0)^a^	72.7 (1.3)^a^	10.1 (1.2)^a^	9.3 (1.5)^a^	40.6 (1.5)^a^	41.9 (1.6)^a^	47.4 (3.2)^a^	49.3 (3.5)^a^
35	50.0 (1.1)^a^	50.0 (1.2)^a^	8.2 (1.3)^a^	7.4 (1.4)^a^	40.2 (1.4)^a^	41.6 (1.6)^a^	98.8 (7.9)^a^	104.3 (7.5)^a^
37	–	–	–	–	–	–	–	–
LSD	4.3	4.5	1.5	1.2	4.8	5.0	6.2	6.5

Similar letters denote insignificant difference between the R and S biotype in each parameter.

*Gmax*: maximum germination percentage; *b*: slope at *X*_50_; *X*_50_: time to 50% of maximum germination at each temperature; *D*_50_: time to 50% germination.

### Osmotic stress assay

**Figure 2:**
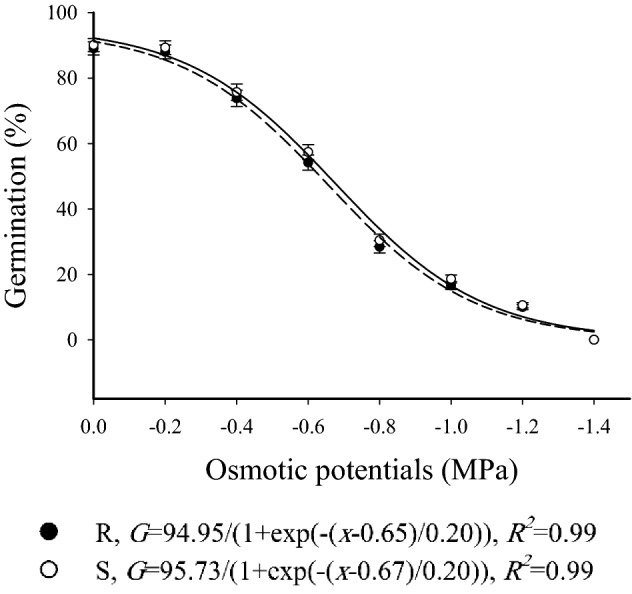
Germination of herbicide-resistant (black circles) and -susceptible (white circles) winter wild oat biotype as affected by different osmotic potentials. The data was fit with a three parameter sigmoidal function using SigmaPlot Version 12.5, from Systat Software, Inc., San Jose California USA, www.systatsoftware.com. The statistical analysis was performed using SAS Version 9 software (SAS Institute Inc., Cary, NC, USA).

The osmotic potentials of 0 (distilled water) and − 0.2 MPa led to the highest germination percentages for the S and R winter wild oat biotypes of winter wild oat. Germination percentages of R and S biotypes at 0 MPa were 90.1 and 89.1%, respectively. These values for − 0.2 MPa were 89.3 and 88.0%, and no difference was observed between these two water potentials. Germination percentage of S and R biotypes decreased from 90.1–89.1% to 10.6–10.3% as osmotic potentials increased, and no germination was observed at − 1.4 MPa. Osmotic potentials of 0, − 0.2, and − 0.4 resulted in the lowest time to 10% germination. The germination response of S and R winter wild oat biotypes to all PEG 6000 solutions over time was similar (Table 3). Also, the germination of both biotypes under all osmotic potentials had a similar trend (Fig. 2).

**Table 3:** Parameter estimates resulted from the fitting of three-parameter sigmoidal function to seed germination data associated with herbicide-resistant (R) and -susceptible (S) winter wild oat biotypes over time under different osmotic potentials. The values in parentheses denote standard error.

Osmotic potential (MPa)	Parameter							
	*Gmax* (%)		*b*		*X*_50_ (h)		*D*_10_ (h)	
	S	R	S	R	S	R	S	R
0 (Distilled water)	90.1 (2.1)^a^	89.1 (1.7)^a^	5.9 (1.0)^a^	5.9 (1.2)^a^	31.9 (1.5)^a^	32.8 (1.3)^a^	19.7 (1.3)^a^	20.5 (1.2)^a^
− 0.2	89.3 (2.1)^a^	88.0 (1.9)^a^	5.9 (0.9)^a^	5.7 (1.3)^a^	32.5 (1.6)^a^	33.2 (1.2)^a^	20.2 (0.9)^a^	21.4 (1.1)^a^
− 0.4	75.7 (1.8)^a^	73.8 (2.0)^a^	6.0 (1.1)^a^	5.9 (1.2)^a^	33.0 (1.2)^a^	33.8 (1.4)^a^	21.7 (0.9)^a^	22.9 (1.0)^a^
− 0.6	57.4 (1.8)^a^	54.2 (2.1)^a^	5.1 (1.4)^a^	5.0 (1.1)^a^	33.9 (1.9)^a^	34.7 (1.3)^a^	25.9 (1.2)^a^	27.3 (1.4)^a^
− 0.8	30.4 (1.8)^a^	28.4 (1.4)^a^	3.8 (0.7)^a^	3.7 (0.8)^a^	33.6 (1.8)^a^	34.1 (1.2)^a^	30.8 (1.1)^a^	31.9 (1.3)^a^
− 1.0	18.6 (1.4)^a^	16.6 (1.5)^a^	3.9 (0.7)^a^	4.0 (1.2)^a^	34.7 (1.3)^a^	35.6 (1.5)^a^	36.1 (1.2)^a^	37.2 (1.0)^a^
− 1.2	10.5 (1.0)^a^	10.3 (0.8)^a^	4.3 (0.7)^a^	4.2 (1.0)^a^	35.7 (1.8)^a^	36.4 (1.2)^a^	48.1 (1.7)^a^	51.3 (1.9)^a^
− 1.4	–	–	–	–	–	–	–	–
LSD	5.8	5.7	1.1	1.2	1.6	1.7	3.1	3.4

Similar letters denote insignificant difference between the R and S biotype in each parameter.

*Gmax*: maximum germination percentage; *b*: slope at *X*_50_; *X*_50_: time to 50% of maximum germination at each temperature; *D*_10_: time to 10% germination.

### Salinity stress assay

**Figure 3:**
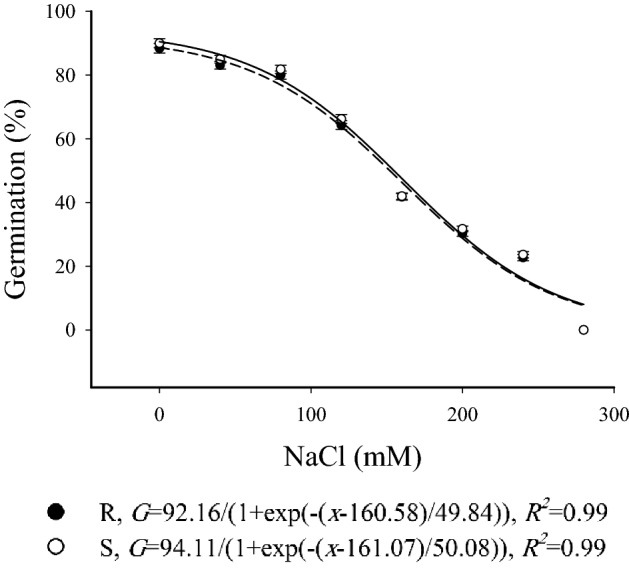
Germination of herbicide-resistant (black circles) and -susceptible (white circles) winter wild oat biotype as affected by different salinities. The data was fit with a three parameter sigmoidal function using SigmaPlot Version 12.5, from Systat Software, Inc., San Jose California USA, www.systatsoftware.com. The statistical analysis was performed using SAS Version 9 software (SAS Institute Inc., Cary, NC, USA).

Increasing concentrations of NaCl from 0 to 240 mM led to a decrease in germination percentage of S and R winter wild oat biotypes from 88.4–90% to 23.6–23.8%. Most of the seeds germinated when subjected to distilled water (0 mM) and 240 mM NaCl. A NaCl concentration of 280 mM led to no germination. The seeds treated with distilled water and 40 mM concentration had the lowest time to 20% germination. Comparison of the germination between S and R biotypes in each osmotic potential showed that there were no differences between the biotypes (Table 4). Moreover, the germination response of the biotypes to increasing NaCl concentrations followed a similar trend (Fig. 3).

**Table 4:** Parameter estimates resulted from the fitting of three-parameter sigmoidal function to seed germination data associated with herbicide-resistant (R) and -susceptible (S) winter wild oat biotypes over time under different salinities. The values in parentheses denote standard error.

NaCl (mM)	Parameter							
	*Gmax* (%)		*b*		*X*_50_ (h)		*D*_20_ (h)	
	S	R	S	R	S	R	S	R
0 (Distilled water)	90.0 (1.7)^a^	88.4 (1.4)^a^	7.6 (1.1)^a^	7.9 (1.2)^a^	33.3 (1.1)^a^	34.3 (1.4)^a^	23.6 (1.8)^a^	24.6 (1.5)^a^
40	85.0 (1.9)^a^	83.1 (1.7)^a^	6.2 (1.0)^a^	6.2 (1.2)^a^	33.8 (1.6)^a^	34.7 (1.5)^a^	26.4 (1.3)^a^	27.5 (1.2)^a^
80	81.9 (1.5)^a^	80.0 (1.7)^a^	5.7 (1.0)^a^	5.7 (1.1)^a^	34.7 (1.3)^a^	35.4 (1.5)^a^	28.3 (1.2)^a^	29.1 (1.1)^a^
120	66.2 (2.1)^a^	64.3 (1.8)^a^	5.6 (1.2)^a^	5.7 (1.0)^a^	34.6 (1.9)^a^	35.7 (1.2)^a^	29.8 (1.4)^a^	31.1 (1.6)^a^
160	42.0 (1.6)^a^	41.8 (1.2)^a^	5.6 (1.1)^a^	5.6 (1.2)^a^	38.4 (1.7)^a^	40.0 (1.5)^a^	37.9 (1.8)^a^	39.5 (1.4)^a^
200	31.7 (1.4)^a^	30.3 (1.3)^a^	5.3 (0.8)^a^	5.2 (1.1)^a^	40.1 (1.8)^a^	41.3 (1.3)^a^	4.9 (1.5)^a^	44.7 (1.3)^a^
240	23.6 (1.1)^a^	22.8 (0.9)^a^	5.1 (0.8)^a^	5.2 (1.0)^a^	39.5 (1.5)^a^	39.9 (1.0)^a^	48.3 (1.8)^a^	50.2 (1.8)^a^
280	–	–	–	–	–	–	–	–
LSD	5.6	5.3	1.2	1.3	1.7	1.9	3.9	4.2

Similar letters denote insignificant difference between the R and S biotype in each parameter.

*Gmax*: maximum germination percentage; *b*: slope at *X*_50_; *X*_50_: time to 50% of maximum germination at each temperature; *D*_20_: time to 20% germination.

### pH assay

**Figure 4:**
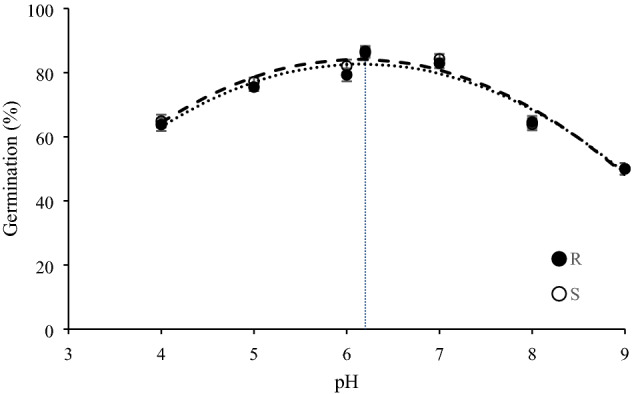
Germination of herbicide-resistant (black circles) and -susceptible (white circles) winter wild oat biotype as affected by different pHs. The dotted vertical line shows the pH of distilled water (pH = 6.2). The data was fit using Microsoft Excel Version 2013 software. The statistical analysis was performed using SAS Version 9 software (SAS Institute Inc., Cary, NC, USA).

Germination percentage in S and R biotypes increased from 64.9–63.8% to 86.7–86.2% percent with increasing pH values and reached its maximum in 6, 6.2, and 7 pH treatments. However, pH values of 8 and 9 led to a decline in germination. The lowest time to 50% germination was observed in 5, 6, 6.2, and 7 pHs, which ranged from 34.2 to 38.6 h. In all pH treatments, no differences in germination between resistant and susceptible biotypes were observed (Table 5). The coefficients of the equation describing the germination response to pH values for S and R biotypes were similar (Fig. 4).

**Table 5:** Parameter estimates resulted from the fitting of three-parameter sigmoidal function to seed germination data associated with herbicide-resistant (R) and -susceptible (S) winter wild oat biotypes over time under different pHs. The values in parentheses denote standard error.

pH	Parameter							
	*Gmax* (%)		*b*		*X*_50_ (h)		*D*_50_ (h)	
	S	R	S	R	S	R	S	R
4	64.9 (1.9)^a^	63.8 (1.2)^a^	7.2 (0.9)^a^	6.9 (1.1)^a^	33.7 (1.3)^a^	34.1 (1.1)^a^	42.5 (2.8)^a^	43.1 (2.4)^a^
5	77.2 (1.8)^a^	75.5 (1.6)^a^	7.2 (1.1)^a^	6.8 (1.1)^a^	33.5 (1.6)^a^	34.0 (1.4)^a^	37.8 (2.4)^a^	38.6 (2.3)^a^
6	82.1 (1.4)^a^	79.3 (1.8)^a^	7.0 (0.8)^a^	6.6 (1.0)^a^	32.8 (1.5)^a^	33.0 (1.4)^a^	35.9 (2.6)^a^	36.5 (2.3)^a^
6.2 (Distilled water)	86.7 (2.0)^a^	86.2 (1.3)^a^	6.6 (0.7)^a^	6.4 (0.7)^a^	32.2 (1.8)^a^	32.1 (1.3)^a^	34.3 (2.2)^a^	34.2 (2.3)^a^
7	84.3 (1.7)^a^	82.9 (1.3)^a^	8.0 (1.0)^a^	7.9 (1.2)^a^	33.9 (1.7)^a^	34.0 (1.3)^a^	36.9 (2.6)^a^	37.3 (2.1)^a^
8	63.9 (1.6)^a^	64.6 (1.3)^a^	6.9 (0.9)^a^	7.3 (1.0)^a^	33.2 (1.7)^a^	34.0 (1.6)^a^	42.0 (2.2)^a^	43.0 (2.4)^a^
9	50.1 (1.3)^a^	50.0 (1.2)^a^	9.3 (0.8)^a^	9.0 (0.8)^a^	33.6 (1.6)^a^	33.8 (1.3)^a^	90.7 (2.7)^a^	93.4 (2.2)^a^
LSD	4.3	4.1	1.1	1.2	2.0	1.7	2.7	3.1

Similar letters denote insignificant difference between the R and S biotype in each parameter.

*Gmax*: maximum germination percentage; *b*: slope at *X*_50_; *X*_50_: time to 50% of maximum germination at each temperature; *D*_50_: time to 50% germination.

### Darkness assay

No differences were observed between the germination percentages of R and S winter wild biotypes under light (87.3–88.3%) and darkness (86.3–87.3%) conditions. Also, time to 50% germination of S and R biotypes was similar under both light and darkness conditions, which ranged from 33.8 to 34.1 h (Table 6).

**Table 6:** Parameter estimates resulted from the fitting of three-parameter sigmoidal function to seed germination data associated with herbicide-resistant (R) and -susceptible (S) winter wild oat biotypes over time under light and darkness conditions. The values in parentheses denote standard error.

Light regime	Parameter					
	*Gmax* (%)		*b*		*X*_50_ (h)	
	S	R	S	R	S	R
Light	88.2 (1.3)^a^	87.3 (1.3)^a^	7.9 (0.9)^a^	7.8 (1.0)^a^	34.1 (1.1)^a^	33.9 (1.2)^a^
Darkness	87.3 (1.2)^a^	86.3 (1.1)^a^	7.8 (0.8)^a^	7.7 (0.9)^a^	33.9 (1.1)^a^	33.8 (0.97 ^a^
LSD	3.3	3.5	1.1	1.2	1.1	1.2

Similar letters denote insignificant difference between the R and S biotype in each parameter.

*Gmax*: maximum germination percentage; *b*: slope at *X*_50_; *X*_50_: time to 50% of maximum germination at each temperature.

### Burial depth assay

**Figure 5:**
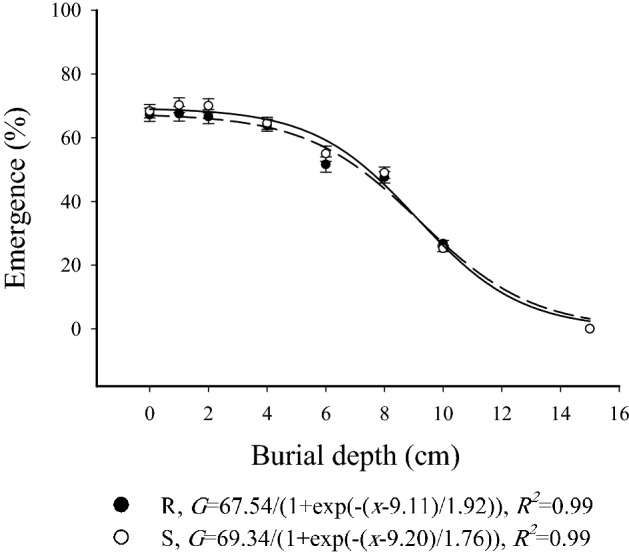
Germination of herbicide-resistant (black circles) and -susceptible (white circles) winter wild oat biotype as affected by different burial depths. The data was fit with a three parameter sigmoidal function using SigmaPlot Version 12.5, from Systat Software, Inc., San Jose California USA, www.systatsoftware.com. The statistical analysis was performed using SAS Version 9 software (SAS Institute Inc., Cary, NC, USA).

**Figure 6:**
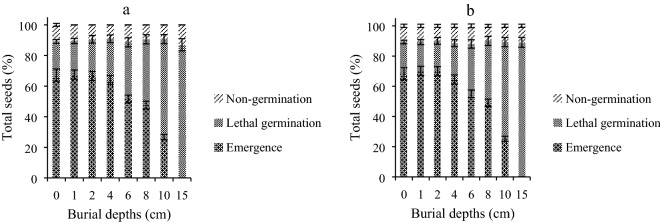
Percentage of the emerged and non-germinated seeds as well as the germinated seeds which failed to reach the soil surface in herbicide-resistant (**A**) and –susceptible (**B**) winter wild oat biotype as affected by different burial depths. The figures were drawn using Microsoft Excel Version 2013 software. The statistical analysis was performed using SAS Version 9 software (SAS Institute Inc., Cary, NC, USA).

The results of seed burial depths showed that the emergence percentage was the highest in 0, 1, and 2 cm treatments (ranging from 66.6 to 70.3%), but it declined thereafter and reached zero when the seeds were buried under 15 cm of soil (Fig. 5). Time to 20% emergence increased from 102.8 to 176.6 h with increasing seed burial depths, and the winter wild oat seeds placed on the soil surface and the ones buried under 1 and 2 cm of soil had a higher emergence rate. No difference was observed between S and R winter wild oat biotypes regarding emergence percentage and rate (Table 7). Also, the percent of the germinated seeds which failed to reach the soil surface increased with increasing burial depth, whereas the depth of seed burial did not affect the percent of non-germinated seeds (Fig. 6). The results of the tetrazolium test showed that the ungerminated seeds were dead.

**Table 7:** Parameter estimates resulted from the fitting of three-parameter sigmoidal function to seed emergence data associated with herbicide-resistant (R) and -susceptible (S) winter wild oat biotypes over time buried under different depths. The values in parentheses denote standard error.

Burial depth (cm)	Parameter							
	*Gmax* (%)		*b*		*X*_50_ (h)		*D*_20_ (h)	
	S	R	S	R	S	R	S	R
0	68.3 (2.1)^a^	67.2 (2.3)^a^	11.2 (0.8)^a^	10.2 (1.1)^a^	112.7 (2.3)^a^	112.9 (1.9)^a^	102.8 (2.9)^a^	104.1 (2.7)^a^
1	70.3 (1.8)^a^	67.5 (1.9)^a^	10.2 (1.6)^a^	9.7 (1.2)^a^	113.5 (2.5)^a^	112.1 (2.1)^a^	104.1 (2.3)^a^	103.7 (2.3)^a^
2	70.0 (1.4)^a^	66.6 (2.3)^a^	11.7 (1.1)^a^	10.2 (1.0)^a^	114.7 (2.5)^a^	113.6 (2.9)^a^	103.9 (2.3)^a^	104.9 (2.6)^a^
4	64.5 (1.7)^a^	63.9 (1.4)^a^	10.7 (1.3)^a^	9.8 (1.2)^a^	115.7 (1.7)^a^	114.7 (1.6)^a^	107.4 (2.5)^a^	106.9 (2.3)^a^
6	55.1 (1.7)^a^	51.6 (1.9)^a^	10.7 (1.3)^a^	9.9 (1.1)^a^	119.2 (2.2)^a^	117.8 (2.1)^a^	113.2 (2.1)^a^	113.3 (2.3)^a^
8	49.3 (1.6)^a^	47.6 (1.2)^a^	12.3 (0.8)^a^	12.3 (0.8)^a^	123.3 (2.6)^a^	124.3 (2.3)^a^	118.6 (2.3)^a^	120.4 (2.6)^a^
10	25.2 (1.2)^a^	26.7 (1.1)^a^	6.5 (0.9)^a^	5.6 (0.8)^a^	167.9 (2.3)^a^	168.8 (2.7)^a^	176.6 (3.2)^a^	174.9 (3.1)^a^
15	–	–	–	–	–	–	–	–
LSD	4.0	3.5	1.2	1.3	2.8	2.7	2.2	1.8

Similar letters denote insignificant difference between the R and S biotype in each parameter.

*Gmax*: maximum germination percentage; *b*: slope at *X*_50_; *X*_50_: time to 50% of maximum germination at each temperature; *D*_20_: time to 20% germination.

## Discussion

According to the results, Ile-2041-Asn mutation was responsible for the resistance to the ACCase inhibitor in winter wild oat from Iran. Various researchers have reported Ile-2041-Asn mutation in the *ACCase* encoding gene as a mechanism of resistance to ACCase inhibiting herbicides, including Papapanagiotou et al.^30^, Du et al.^19,32^, Shergill et al.^33^, Anthimidou et al.^34^, etc.

Asn-2041-Ile mutation in the *ACCase* encoding gene did not affect the germination response of winter wild oat to various temperatures, and germination percentage and rate of the resistant biotype was similar to the susceptible one in all temperature treatments. This is in accordance with the results reported by Travlos^22^, in which no difference was observed between the resistant and susceptible winter wild oat populations when incubated at various temperature regimes. However, *ACCase*-resistant Asia Minor bluegrass (*Polypogon fugax* Nees ex Steud.) biotype had a lower germination percentage compared with the susceptible biotype. The authors pertained the lower germination of resistant biotypes to the fitness cost imposed by Asn-2041-Ile mutation^35^, which is in contrast with the findings of the present study.

The germination response of resistant and susceptible biotypes of wild oat under various osmotic potentials was similar. Both biotypes were able to germinate in a wide range of water potentials, indicating the ability of winter wild oat to infest croplands located in different climatic conditions from arid to humid. Water stress is shown to have negative effects on the germination of many weed species such as junglerice (*Echinochloa colona* (L.) Link)^36^, sourgrass (*Digitaria insularis*)^17^, Tausch's goatgrass (*Aegilops tauschii* Coss.)^37^, etc. Wu et al.^15^ reported that the germination of fenoxaprop-P ethyl-resistant Japanese foxtail biotypes with Trp-1999-Ser mutation was similar to the susceptible biotype under various water stress conditions. Conversely, germination of a glyphosate-resistant Italian ryegrass population plummeted far more than that of the susceptible population, indicating a fitness cost^38^.

Different NaCl solutions had similar effects on the germination of resistant and susceptible winter wild oat biotypes. Although germination was lower in high concentrations of NaCl, both biotypes were able to germinate over a wide range of NaCl concentrations. Salinity has adverse effects on plant germination, and the ability of germination in saline conditions is highly decisive for the establishment of weed species^39^. Du et al.^19^ reported that the germination of *ACCase*-resistant American slough grass biotypes under various salinity conditions varied depending on the mutation on the *ACCase* encoding gene. The biotype with Ile-1781-Leu mutation had a higher germination percentage compared to the susceptible biotype, whereas Trp-2027-Cys and Asn-2041-Ile mutations did not affect germination percentage.

Germination percentage and rate of R winter wild oat biotype were similar to the S biotype. According to the results, both biotypes were able to germinate in a wide range of pH treatments. Species such as texasweed (*Caperonia palustris* (L.) A. St.-Hil.)^40^, giant sensitive plant (*Mimosa diplotricha* C. Wright ex Sauvalle)^41^, American slough grass^19^, etc., have been reported to germinate over a wide pH range. For instance, resistant and susceptible American slough grass biotypes, carrying the Ile-1781-Leu, Trp-2027-Cys and Asn-2041-Ile mutations, showed no difference in germination under different pH treatments^19^. However, ACCase-resistant Asia Minor bluegrass population had a lower germination percentage compared to the susceptible one at 4–10 pH levels^35^. Conversely, glyphosate resistance in an Italian ryegrass population resulted in the enhancement of germination compared to the susceptible population at a pH range of 4–7, indicating a positive fitness^38^.

The ACCase resistant and susceptible winter wild oat biotypes germinated similarly under both dark and light conditions, indicating a lack of fitness cost as a result of Asn-2041-Ile mutation. This was also the case for the glyphosate-resistant junglerice, which showed no significant difference with the susceptible population regarding germination under light and dark conditions^42^. Conversely, the difference between glyphosate-resistant and -susceptible biotypes of sourgrass was more pronounced under light conditions in comparison to darkness^17^.

Percentage and rate of emergence for both S and R winter wild oat biotypes were almost similar in all burial depths. The biotypes failed to emerge from 15 cm depth. Also, the percent of the seedlings which failed to reach the soil surface increased immensely with increasing burial depth. Thus, the adoption of deep tillage may prove helpful in the reduction of emergence in both resistant and susceptible biotypes. Lack of difference in the emergence of glyphosate-resistant and susceptible junglerice populations in different burial depths was reported by Sheng et al.^42^. Glyphosate-resistant sourgrass biotypes had a higher emergence percentage compared to the susceptible biotypes when buried under various soil depths^17^, whereas the emergence percentage of an Asia Minor bluegrass biotype with Ile-2041-Asn mutation was higher than that of the susceptible biotype in different burial depth treatments^35^.

In overall, Ile-2041-Asn mutation imposes no fitness cost on winter wild oat because germination under various environmental conditions including temperature, drought, and salinity stresses, pH, darkness, and emergence from various soil depths was similar between R and S biotypes. Therefore, the same weed management methods used for susceptible populations apply to the resistant ones. In most cases, fitness cost will only express under certain environmental conditions. Therefore, investigation of germination under different environmental field conditions may lead to more realistic results^2^.

It must be noted that other non-resistance related genetic differences were not taken into account in our study, which would also impose a further fitness cost on winter wild oat. Therefore, the present study may have underestimated the fitness costs^43^. Nonetheless, from an ecological point of view, negligible or no fitness cost will lead to more rapid evolution of resistance in the population^44^, and this further emphasizes the importance of controlling winter wild oats with Asn-2041-Ile mutation.

Since no fitness cost at the seed level was observed in the present study, management of the resistant weeds will be more difficult, as mentioned above. Consecutive application of ACCase herbicides will lead to a further increase in the frequency of the resistance alleles^4^. Moreover, no other herbicides may be available for control of winter wild oat in oilseed rape fields in many countries, including Iran. Thus, adopting crop and herbicide rotation is crucial to delay further development of resistance in these species. Also, the researchers are encouraged to test and register new herbicides for control of winter wild oat in oilseed rape fields.

## Materials and methods

### Plant material

The experiments were conducted using the seeds of an herbicide-resistant (R) and a susceptible (S) winter wild oat biotype. The resistance of R seeds to haloxyfop-R methyl ester, clodinafop propargyl, and sethoxydim herbicides had been confirmed previously^31^.

### Sequencing of the *ACCase* gene

The seeds of the resistant and susceptible winter wild biotypes were pre-chilled for 72 h at 4 °C to obtain uniform germination and were then incubated at 25 °C temperature for 24 h^45^. Then, ten pre-germinated seeds of each biotype were sown in 25 cm diameter pots (with a total volume of 900 cm^3^) containing 20 cm of silty-loam soil in a greenhouse. Each pot served as a replicate. Young leaf tissue of the plants (three plants from each R and S biotypes) was sampled at the 3–4 leaf stage. The samples were taken from individual specimens and were kept at − 80° C until the beginning of the experiment. Doyle and Doyle^46^ protocol was used to isolate DNA of the R and S biotypes. Then, the extracted DNA was purified to be sequenced. A mix was prepared for each biotype which included 5 μL Taq DNA Polymerase 2× Master Mix Red (Ampliqon, Denmark), 0.5 μL for each forward and reverse primer, 0.3 μL MgCl2 1.5 mM, 3 μL double distilled water, and 1 μL DNA from each biotype. Then, the mixes were placed in a thermocycler (Lab cycler, Germany) for the chain reaction to begin. PCR was conducted using the primers shown in Table 1^47^. The program started with four minutes of initial denaturation at 94 °C followed by 35 cycles including 30 s at 90 °C, 30 s at 65/60 °C for 1781/1999-2096, respectively, and one minute at 72 °C. The final extension was performed for ten minutes at 72 °C. Then, the PCR product was sent to Bioneer, South Korea for sequencing. The alignment was done using MultAlin software^48^.

### Control of genetic background

The two segregating genotype progenies used in the fitness experiments were derived from a plant that contained the heterozygous Ile-2041-Asn mutation and no other known ACCase mutation. Pairwise comparisons were performed for each mutation between each homozygous resistant and susceptible progenies. Both progenies shared a common genetic background, except for the Ile-2041-Asn mutation. Each parent plant was cultivated appropriately and isolated within a pollen proof enclosure. F_1_ progeny seeds from each plant were collected. Then, homozygous resistant and susceptible plants in the F_1_ progeny were identified by ACCase genotyping, and each was grown in a pollen proof enclosure to produce F_2_ seeds. These seeds were used for subsequent experiments, which were started immediately.

### Seed pre-treatment

To eliminate the seed dormancy, the hulled seeds of R and S biotypes were placed in 12 cm Petri dishes topped with Whatman No. 1 paper. Then, 8 ml of distilled water was added to the Petri dishes, which were then kept in a refrigerator at 4 °C for 72 h^43^.

### General germination test protocol

All experiments were conducted for R and S winter wild biotypes separately based on a completely randomized design with four replications. Twenty-five pre-treated seeds were placed in 9 cm Petri dishes topped with Whatman No. 1 paper. Each Petri dish served as one replicate. Five mL of distilled water or other solutions (see below) were added to each Petri dish, which was then transferred into an incubator with no lights. The Petri dishes were covered with plastic freezers to prevent the evaporation of the solutions and were inspected 2–6 times a day depending on germination rate for 10 days. The seeds were considered as germinated when the radicle became visible. All experiments were conducted twice.

### Temperature assay

The Petri dishes were incubated at 5, 10, 15, 20, 25, 30, 35, and 37 °C (since no germination was observed at 40 °C, the seeds were also subjected to 37 °C). Other conditions were the same as mentioned in the General germination test protocol section. The temperature which resulted in the highest germination was used to conduct other assays^49^.

### Osmotic stress assay

Polyethylene glycol 6000 (PEG 6000) solutions prepared as described by Michel and Kaufmann^50^ were added to the Petri dishes at 0 (distilled water), − 0.2, − 0.4, − 0.6, − 0.8, − 1.0, − 1.2 and − 1.4 MPa osmotic potentials. Then, the seeds were incubated at the temperature which resulted in the highest germination (see *Temperature assay* section). Other conditions were the same as mentioned in the General germination test protocol section.

### Salinity stress assay

The seeds were subjected to NaCl concentrations of 0 (distilled water), 40, 80, 120, 160, 200, 240, and 280 mM at the temperature which resulted in the highest germination (see *Temperature assay* section). Other conditions were the same as mentioned in the General germination test protocol section.

### pH assay

The method presented by Susko et al.^51^ was used for this experiment. Distilled water (pH = 6.2) and pH solutions with 4, 5, 6, 7, 8, and 9 values were added to the Petri dishes. The Petri dishes were then incubated at the temperature which resulted in the highest germination (see *Temperature assay* section). Other conditions were the same as mentioned in the General germination test protocol section.

### Darkness assay

The seeds were placed in Petri dishes in a dark room under green light^52^. Then, the Petri dishes were wrapped in two layers of aluminium foil and incubated at the temperature which resulted in the highest germination (see *Temperature assay* section). The inspection of seeds was also done in the darkness under green light. Other conditions were the same as mentioned in the General germination test protocol section.

### Burial depth assay

The experiment was conducted based on a completely randomized design with three replications at the greenhouse. The greenhouse temperature was 22/16 °C (day/night) with a 12/12 h of light/darkness. The pots were filled with a certain amount of silty-loam soil. Then twenty-five seeds from the R and S biotypes were placed on the surface of the soil in plastic pots. The pots were then filled with the same type of soil to obtain the burial depth of 0, 1, 2, 4, 6, 8, 10, and 15 cm. Irrigation of the pots was done using a sprayer. The seedling was considered as emerged upon the observation of epicotyl at the soil surface. The pots were inspected every 12 h to record the emergence, which was finished when no further emergence was observed (72 h). The pots which had non-emerged plants were examined at the end of the test to see whether the seeds did not germinate or the epicotyl was unable to reach the soil surface. The seeds which were unable to germinate were subjected to the tetrazolium (TZ) test to determine their viability.

### Statistical analysis

Since no interaction was observed between the treatments and the experimental runs, the data from the two experimental runs were pooled. Three parameter sigmoidal function (Eq. 1) was fitted to the data associated with cumulative germination percentage the mentioned environmental conditions over time:

1 $$G = Gmax/\left( {1 + \exp \left( { - \left( {T - X_{50} } \right)/b} \right)} \right)$$

where *G* is the percent of seeds germinated at time *T*, *Gmax* is maximum germination percentage, *X*_50_ is the time to 50% maximum seed germination, and b is the slope of the curve at *X*_50_. The time to 10%, 20%, and 50% germination (*D*_10_, *D*_20*,*_ and *D*_50_) was also determined by interpolation when necessary.

Equation 1 was also used to describe the maximum germination percentage data against various osmotic and salinity potentials and burial depths. A quadratic function was fitted to the data associated with a maximum germination percentage over various pHs. A t-test was conducted to compare the coefficients of the R and S pH curves.

Comparison of means for the parameters within and between the biotypes was done using the LSD method and t-test at *p* < 0.05, respectively. Analysis of variance and regression as well as the drawing of figures were done using SigmaPlot Version 12.5 (Systat Software, San Jose, CA), SAS Version 9 (SAS Institute Inc., Cary, NC, USA), and Microsoft Excel Version 2013 softwares.

## Conclusion

The evolution of resistance to ACCase inhibitors in winter wild oat due to Ile-2041-Asn mutation was not associated with a fitness cost at the seed level, as there was no difference between the germination response of S and R winter wild oat under various environmental conditions including drought, salinity, pH and light, and darkness. Also, S and R plants showed similar percentages of emergence, lethal germination, and non-germination under different burial depths. Lack of fitness cost indicates that the seeds of both S and R winter wild oat will germinate and emerge similarly under the studied environmental conditions in the absence of ACCase-inhibiting herbicide selection pressure.

## Data Availability

The datasets generated during and/or analyzed during the current study are available from the corresponding author on reasonable request.
